# Prediction Models for Perioperative Blood Transfusion in Patients Undergoing Gynecologic Surgery: A Systematic Review

**DOI:** 10.3390/diagnostics14182018

**Published:** 2024-09-12

**Authors:** Zhongmian Pan, Kittipat Charoenkwan

**Affiliations:** 1 Department of Obstetrics and Gynecology, Faculty of Medicine, Chiang Mai University, Chiang Mai 50200, Thailand; panzhongmian7101@163.com; 2 Department of Obstetrics and Gynecology, Faculty of Medicine, Affiliated Hospital of Youjiang Medical University for Nationalities, Baise 533000, China

**Keywords:** blood loss, blood transfusion, gynecologic surgery, prediction model, systematic review

## Abstract

This systematic review aimed to evaluate prediction models for perioperative blood transfusion in patients undergoing gynecologic surgery. Given the inherent risks associated with blood transfusion and the critical need for accurate prediction, this study identified and assessed models based on their development, validation, and predictive performance. The review included five studies encompassing various surgical procedures and approaches. Predicting factors commonly used across these models included preoperative hematocrit, race, surgical route, and uterine fibroid characteristics. However, the review highlighted significant variability in the definition of perioperative periods, a lack of standardization in transfusion criteria, and a high risk of bias in most models due to methodological issues, such as a low number of events per variable, inappropriate handling of continuous and categorical predictors, inappropriate handling of missing data, improper methods of predictor selection, inappropriate measurement methods for model performance, and inadequate evaluations of model overfitting and optimism in model performance. Despite some models demonstrating good discrimination and calibration, the overall quality and external validation of these models were limited. Consequently, there is a clear need for more robust and externally validated models to improve clinical decision-making and patient outcomes in gynecologic surgery. Future research should focus on refining these models, incorporating rigorous validation, and adhering to standardized reporting practices.

## 1. Introduction

Every year, 313 million surgeries are performed worldwide [1]. Gynecological surgery is a common procedure, involving methods such as laparoscopy and open surgery. Due to the significant number of pelvic blood vessels and their concealed location, gynecological surgeries often come with complications such as intraoperative and postoperative bleeding. Blood transfusion is a life-saving measure when necessary. There are varying risks associated with blood transfusion in gynecological surgery. The incidence of blood transfusion in minimally invasive surgery patients is 1.7%, while open surgery can reach up to 22.4% [2]. In high-income countries, blood transfusion is most commonly used in cardiovascular and transplant surgeries, large-scale trauma, and supportive treatment for solid tumors and hematological malignancies [3]. Proper and adequate blood transfusion treatment is essential for patient safety. However, errors in clinical judgment can lead to underuse or overuse of blood, resulting in wastage of blood products and complications. These complications may include transfusion reactions, hemolysis, transfusion-associated circulatory overload, and transfusion-related acute lung injury. In severe cases, these complications can result in pulmonary edema and even death [4,5]. Infusing blood that has been stored for a prolonged period can lead to the presence of non-transferrin-bound iron in the circulation. This can result in increased transfusion-related complications, such as infections and prolonged patient hospitalization [6]. These chaotic conditions may lead to a shortage of blood and exacerbate the strain on blood resources. Consequently, restrictive transfusion strategies have been proposed, which have been shown to be as effective as free transfusion strategies [7,8].

It is important to accurately predict which patients undergoing surgery will urgently need perioperative blood transfusions. By focusing on the preparation and use of blood components, we can improve efficiency, reduce costs, and minimize potential adverse reactions caused by transfusions. This highlights the need for the development of an accurate prediction model [9]. Over the years, researchers have dedicated themselves to developing various prediction models, utilizing traditional statistical methods as well as, more recently, artificial intelligence and machine learning. A prediction model is a mathematical equation that generates personalized models by analyzing complex data structures. These models are designed to improve individuals’ health status and enhance clinical decision-making by aiding in diagnosis, prognosis, monitoring, and treatment [10,11]. The prediction of blood transfusion risk is closely linked to the prediction of blood loss, and strategies based on patient blood management are propelling the advancement of blood loss and transfusion prediction models [12,13,14,15,16,17,18].

We conducted this systematic review to identify and assess the quality and predictive performance of all prediction models for perioperative blood transfusion associated with gynecologic surgery. At the same time, we identified and summarized common predictive factors for perioperative blood transfusion.

## 2. Materials and Methods

We conducted a systematic review of preoperative prognostic models that predict perioperative blood transfusion in women undergoing gynecologic surgery according to the guidelines provided by Debray et al. [19]. The review protocol was prospectively registered in PROSPERO, the international prospective register of systematic reviews (registration number CRD42024518545). We report this review following the transparent reporting of multivariable prediction models for individual prognosis or diagnosis: the checklist for systematic reviews and meta-analyses (TRIPOD-SRMA) [20].

### 2.1. Eligibility Criteria

The eligibility criteria for primary research studies to be considered for inclusion in this review were based on the population, intervention, comparator, outcome(s), timing, and setting (PICOTS) framework. Studies were considered if they met the following inclusion criteria: Population, Intervention, Comparator, Outcome, Timing, and Setting.

#### 2.1.1. Population/Participants

The study population comprised patients undergoing elective gynecological surgery, including those having surgery for benign or malignant tumors. Surgical methods were diverse, including open surgery, laparoscopic surgery, hysteroscopic surgery, robotic surgery, etc. These criteria excluded patients undergoing emergency surgery.

#### 2.1.2. Interventions and Comparators (Models)

Related models included multivariable risk prediction models using traditional statistical and/or machine learning methods to develop and validate predictions for perioperative blood transfusion. The aims of the eligible studies included prediction model development with internal validation (with or without external validation) and external model validation (with or without model updating). Studies that reported model development without any form of validation were excluded from the analysis. Studies reporting the impact assessment of a prognostic model were not included. There were no restrictions on the types of variables included in the model, such as demographic data, preoperative indicators, pathology, imaging, biomarkers, etc.

#### 2.1.3. Outcomes

Perioperative blood transfusion, a binary categorical variable, was defined as the transfusion of donor blood during or after surgery.

#### 2.1.4. Timing

We focused on the models applied preoperatively to predict perioperative blood transfusion during or following the operation.

#### 2.1.5. Setting

In our review, we concentrated on women undergoing elective major gynecologic surgery. We aimed to optimize clinical care by ensuring timely blood product orders and minimizing blood wastage for low-risk patients.

### 2.2. Information Sources and Search Strategy

We conducted a comprehensive electronic database search to identify relevant studies, including MEDLINE and EMBASE. Our search utilized the terms “gynecological surgery”, “prediction models”, and “blood loss/transfusion”. We employed Boolean operators to customize the search strategies for each database to studies published in English from its inception until March 2024 (File S1). This method ensured a broad and thorough retrieval of relevant studies. In addition, we thoroughly searched the citation lists of included studies, relevant publications, systematic reviews, and review articles to capture any supplementary studies not indexed in the primary databases. To streamline the literature review process, we utilized EndNote reference management software Version 21.2 [21], which helped us organize and record the selection decisions, ultimately enhancing efficiency and accuracy.

### 2.3. Study Selection

The study selection process was carefully documented using the preferred reporting items for systematic reviews and meta-analyses (PRISMA) flow diagram [22]. Both authors conducted the study selection. Initially, duplicate records identified in the combined search results were removed using EndNote reference management software Version 21.2. The data were then uploaded to ASReview Lab software Version 1.6 [23], developed at Utrecht University, for screening of titles and abstracts. The titles and abstracts of the retrieved articles were screened for relevance, and those clearly irrelevant were discarded. Subsequently, full-text articles of potentially eligible studies were obtained and thoroughly examined by the two authors independently to determine if they met the inclusion criteria. Reasons for exclusion were systematically recorded. Any disagreements between the authors during the selection process were resolved through discussion.

### 2.4. Data Collection

Data were extracted by ZP using a standardized data extraction form developed according to the critical appraisal and data extraction for systematic reviews of prediction modelling studies (CHARMS) checklist [24] and rechecked by KC. Disagreements were resolved through discussion. The data extraction form is provided as a Supplementary Material (File S2).

### 2.5. Risk of Bias Assessment

The two authors independently evaluated the risk of bias of each model reported in the included studies using the prediction model risk of bias assessment tool (PROBAST) [25,26]. Any differences were resolved through discussion. The PROBAST tool evaluates the risk of bias (ROB) and the concerns regarding the applicability of primary research in developing or validating multivariable prediction models for diagnosis or prognosis. It examines four key areas of predictive model research: participants, predictors, outcomes, and analysis. Based on the ROB classification for each domain, the authors determined whether the overall ROB of the prediction model is low, high, or unclear. The tool classifies the applicability of the first three areas as low, high, or unclear. An Excel template for extracting data and assessing the risk of bias and the applicability of prediction models developed by Fernandez-Felix et al. was used [27]. ROB and applicability assessment charts were employed to illustrate each model’s ROB and applicability.

### 2.6. Summary Measures of the Model’s Predictive Performance

Our analysis focused on two statistical measures that gauge the model’s predictive performance: discrimination and calibration.

Discrimination refers to how well a prediction model can differentiate between subjects who will experience the outcome and those who will not. We extracted the concordance (c)-statistic, which represents the estimated likelihood that, for any pair of subjects where one experiences the outcome and the other does not, the predicted risk of an event is higher for the former. If the c-statistic was not reported, we planned to calculate it from other reported information or extract its variations, including the area under the receiver operating characteristics curve (AUC-ROC) or Somer’s D statistic, if available [28].

Calibration involves assessing how accurately a model predicts the likelihood of an event happening, such as a perioperative blood transfusion. This is often shown visually in calibration plots, which compare the model’s predicted probabilities against the actual frequencies of the event in the validation dataset. We collected calibration assessment data for each model in the studies, including calibration plots with calibration intercepts or calibration slope measures, observed-to-expected event ratios, and Hosmer-Lemeshow (HL) tests [28].

### 2.7. Visualization

Bar charts illustrating predictor prevalence and risk of bias summary were created using the ‘dplyr’, ‘reshape2’, and ‘ggplot2’ packages of the R statistical software Version 4.4.1 [29,30,31,32].

## 3. Results

### 3.1. Results of the Search

**Figure 1:**
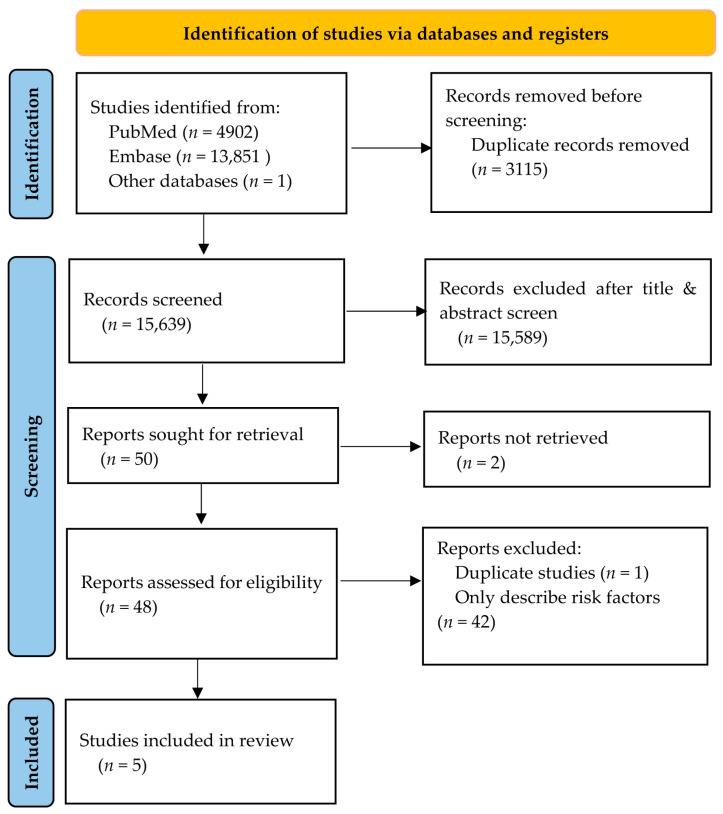
PRISMA flow diagram.

Initially, a total of 18,754 studies were screened. After selecting the titles and abstracts, 50 articles were qualified. During the full-text screening, 45 studies were excluded for the following reasons: two did not have a full text, one had completely duplicated content with the other, and 42 only described risk factors without involving the development and/or validation of prediction models. This systematic review ultimately included five studies. Four of the five included studies were retrospective cohort studies. One study was a retrospective case-control study. The characteristics of these included studies are presented in Table 1. Figure 1 illustrates the study selection process using the PRISMA flow diagram.

### 3.2. Included Studies

#### 3.2.1. Participants

The five included studies involved 39,770 participants. 32,204 participants were used to develop and internally validate the model, and 7566 participants were included in the external validation of the model. Except for two studies from hospitals in the United States (18,415 patients) [33,36], the data from the other three studies were from the American College of Surgeons (ACS) National Surgical Quality Improvement Program (NSQIP) database (21,355 patients) [34,35,37]. The characteristics of participants in the included studies in this review vary greatly, with participants ranging in age from their early 30s to over 65 years old. Among the five studies, three were conducted on patients who underwent myomectomy for uterine fibroids [35,36,37], and one focused on laparoscopic myomectomy surgery for uterine fibroids [37]. There was a study on hysterectomies for ovarian cancer [34], and another study included all patients who underwent gynecological surgery [33], including benign and malignant surgical patients. Regarding the surgical route, one study only focused on laparoscopic surgery [37], two studies distinguished patients between laparoscopic and traditional open surgery [34,35], and the other two studies included all types of surgeries, such as laparoscopy, open surgery, robotic surgery, vaginal surgery, and hysteroscopy, without detailed classification [33,36].

#### 3.2.2. Predictors

**Figure 2:**
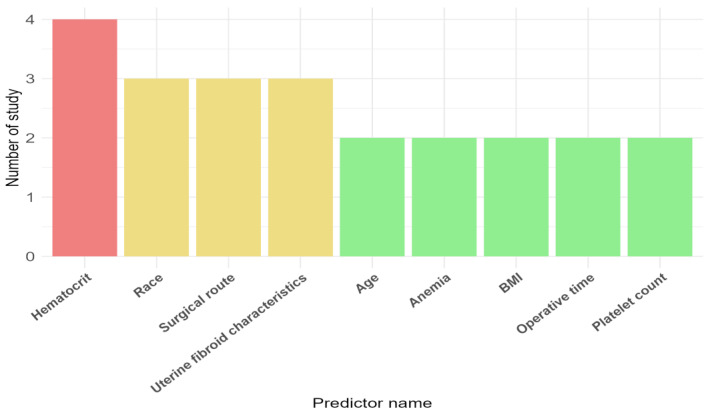
The top nine prevalent predictors for blood transfusion-related outcomes included in the final developed model. The numbers are out of 23 predictors reported. BMI, body mass index.

Table 2 summarizes the characteristics of the models assessed in the included studies. All studies reported the predicting factors selected in the model, and in each study developing the model, 10 to 36 candidate predictive factors were considered, including age, body mass index (BMI), race, pregnancy and childbirth history, disease history, surgical history, preoperative features (including preoperative hemoglobin, preoperative platelet count, and hematocrit), intraoperative features (including surgical method, surgical time, disease type, and cancer staging), and postoperative pathology (including specimen weight, tumor number, tumor diameter, etc.). The final model includes 4 to 12 predictive factors. The most common predictive factors in the final model are preoperative hematocrit (*n* = 4/5, 80%), race (*n* = 3/5, 60%), surgical route (*n* = 3/5, 60%), and uterine fibroid characteristics (*n* = 3/5, 60%) (Figure 2).

#### 3.2.3. Outcome

The outcomes were perioperative blood transfusion requirements. All included studies have developed and validated binary transfusion outcome models. Different definitions were given in the studies to predict blood transfusion during the perioperative period. Three studies defined the perioperative period as within 30 days after surgery [33,34,35], one study defined it as within 24 h after surgery [36], and one study defined it as within 72 h after surgery [37]. For patients who required perioperative blood transfusion, no studies have reported transfusion indications (such as the degree of hemoglobin decrease or intraoperative blood loss) or methods for calculating blood loss.

#### 3.2.4. Prediction Model Development and Validation

In the five included studies, six models were developed and validated. In Hamilton et al. [37], two models were developed. Logistic regression was the most commonly used method [33,34,35,37]. In Walczak et al. [36], artificial neural networks (ANNs) were employed as the modeling method. Regarding model validation, four studies underwent internal validation, with three studies using bootstrapping [33,35,37] and one study using cross-validation techniques [36]. In one study, internal validation was not conducted [34]. Only two models underwent external validation [33,34], with 6100 and 1466 participants, respectively, using temporal validation as the validation method. The external validation model did not provide the number of resulting events that occurred. An external validation study mentioned missing data and used multiple imputation [33]. The externally validated c-statistics were 0.92 [33] and 0.69 [34], respectively. This highlights the limited practice of testing models in different groups to evaluate their universality and reliability. Among all studies, two studies used the c-statistic to represent the model’s predictive performance [33,34]. Three models reported other performance metrics, including classification metrics such as sensitivity, specificity, and accuracy [36] and AUC [35,37]. Interestingly, three studies [34,35,37] established a preoperative transfusion risk scoring system.

### 3.3. Risk of Bias and Applicability Concerns

**Figure 3:**
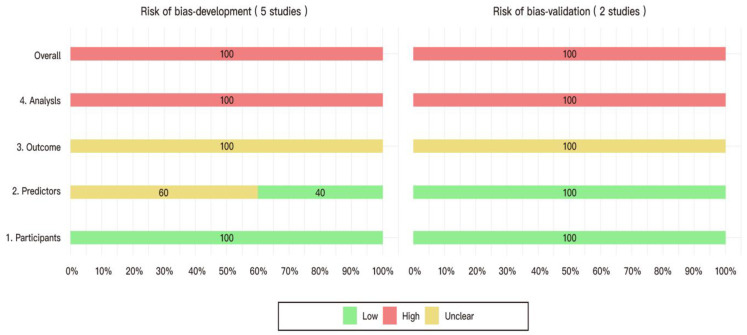
Bar chart summarizing the risk of bias of all models developed and validated in the included studies.

Table 3 summarizes the results of the risk of bias and applicability concerns assessment of the included model development studies according to PROBAST. Figure 3 provides a domain-based summary of the risk of bias assessment. The Supplementary Material (File S3) section presents a detailed risk of bias assessments for each model development and validation analysis.

#### 3.3.1. Model Development

##### Participants

All models were considered to have a low risk of bias and low concern for applicability. The participants’ characteristics and settings were relevant to the review questions.

##### Predictors

The risk of bias was considered unclear in the models presented in the three studies. In Klebanoff et al. [35], outcome information was available when assessing predictors due to the case-control study design. In Walczak et al. [36], the number, location, and type of uterine fibroid were selected as candidate predictors. It is not clear whether the information available only during surgery was also used in addition to the information available before surgery when developing the model. The information available during surgery would not be accessible at the time the model was intended to be used for prediction. Similarly, in Hamilton et al. [37], two predictors (large uterus and prolonged operation time) of the 6-predictor model were only available postoperatively. The risk of bias in all other models was low. All models are considered to have low concern for applicability, indicating consistency between the definition, evaluation, or timing of predictor variables and the review questions.

##### Outcome

Although all studies used prespecified outcome definitions of perioperative blood transfusion, no studies reported transfusion indications. Consequently, there was inadequate information to determine the appropriateness of outcome determination, the exclusion of predictors from the outcome definition, uniformity in outcome determination, and independence in determining the outcome from knowledge of predictor information. Therefore, all models were considered to have an unclear risk of bias and an unclear concern for applicability in this domain.

##### Analysis

All models were considered to have a high risk of bias for various reasons, including a low number of events per variable (EPV), inappropriate handling of continuous and categorical predictors, incomplete inclusion of enrolled participants in the analysis, inappropriate handling of missing data, improper method of predictor selection, inappropriate measurement method for model performance, and inadequate evaluation of model overfitting and optimism in model performance.

##### Overall

The models were considered to have a high overall risk of bias according to the guidelines provided with PROBAST. This was due to a high risk of bias in the analysis domain and one or more domains with an unclear risk of bias. Additionally, there was an unclear concern for applicability overall, with an unclear concern noted in the outcome domain and low concerns in all other domains.

#### 3.3.2. Model External Validation

Two models, developed by Stanhiser et al. [33] and Ackroyd et al. [34], underwent external validation using temporal validation as the validation method. The risk of bias assessment results was consistent with those obtained from the assessment of the model development phase.

### 3.4. Models’ Predictive Performances (Table 2)

Of the five studies included, four (80%) provided discrimination information. Two studies reported c-statistic [33,34], while two studies reported AUC-ROC [35,37]. It is generally indicated that values of <0.7 represent poor discrimination, 0.7–0.8 depict acceptable discrimination, and >0.8 signify excellent discrimination.

Of the five studies included, three (60%) provided calibration information through calibration plots and/or HL tests. For the HL test, it is generally indicated that the low *p*-value of ≤0.05 represents poor calibration (a significant mismatch between predicted and observed outcomes), and the high *p*-value of >0.05 signifies good calibration (a close match between the predicted probabilities and the observed outcomes).

Stanhiser et al. [33] showed excellent discrimination of their model in both internal and external validation settings. In addition, the calibration plot showed excellent predictions throughout the range of predicted risks and was accurate through a range of predicted probabilities ranging from 0% to approximately 40% risk of transfusion. Excellent predictive performance was also indicated by the low Brier score of 0.017. In Ackroyd et al. [34], discrimination was considered highly acceptable in the model development setting but less satisfactory in the external validation setting. A strong agreement was found between the predicted and actual probabilities of blood transfusion across different risk scores. Furthermore, both the development and validation sets showed good calibration, with high HL test *p*-values of 0.81 and 0.56, respectively. Similarly, Klebanoff et al. [35] demonstrated highly acceptable discrimination of their developed model in internal validation. Also, good calibration was demonstrated by the validation model’s calibration plot and a high HL test *p*-value of 0.68 for the model development set. In Hamilton et al. [37], two models were proposed, wherein the 6-parameter model showed significantly better discrimination compared to the 4-parameter model.

In the study by Walczak et al. [36], the effectiveness of using artificial neural networks (ANNs) to predict the estimated blood loss (EBL) and transfusion requirements of 96 myomectomy patients at a single institution was examined. Backpropagation and radial basis function ANN models were created to predict EBL and perioperative transfusion needs, along with a regression model. The single hidden layer backpropagation ANN models performed the best for both prediction tasks. EBL was predicted within an average of 127.33 mL of measured blood loss, and transfusions were predicted with 71.4% sensitivity and 85.4% specificity. Furthermore, a combined ANN ensemble model using the output of the EBL ANN as an input variable to the transfusion prediction ANN was developed, resulting in 100% sensitivity and 62.9% specificity.

## 4. Discussion

### 4.1. Summary of Findings

We conducted a systematic review to identify studies addressing the development and validation of preoperative prediction models for perioperative blood transfusion in gynecologic surgery patients, and to examine their specific characteristics, assess bias risk, and summarize the performance of the proposed models. Five observational studies involving 39,770 participants were included [33,34,35,36,37]. There were significant variations among studies regarding participant characteristics, gynecologic conditions being treated, surgical procedures, and surgical approaches. Also, the numbers of predicting factors included in the initial (10–36 predictors) and the final models (4–12 predictors) were different among the studies. Preoperative hematocrit, race, surgical route, and uterine fibroid characteristics were the top four predictors incorporated among all the models. Regarding the outcome, while all studies aimed to predict perioperative blood transfusion, different definitions for the end of the ‘perioperative period’ were given, ranging from 24 h to 30 days. Importantly, no studies have reported transfusion indications. Logistic regression was the most commonly employed method of prediction model development, with one study proposing the ANN, one of the machine learning algorithms [36].

We rated all included studies as having a high risk of bias, primarily resulting from the high risk of bias in the Analysis domain of the PROBAST criteria. The insufficient number of participants with the outcome, inappropriate handling of continuous and categorical predictors, inappropriate handling of missing data, improper method of predictor selection, and lack of model internal validation were methodological concerns encountered in the included studies. These factors heighten the likelihood of overfitting models and skewing performance estimates. In addition, the risk of bias in the Outcome domain was rated as unclear for all studies. The lack of clearly specified criteria for blood transfusions led to limited assessment of the appropriateness of outcome determination and the proper exclusion of predictors from the outcome definition. The difference in the specified time intervals of the ‘perioperative period’ further complicated the comparison between the studies. The longer (30 days) postoperative interval employed in three out of five studies also brought into question the possible influence of other postoperative factors on the need for blood transfusion and the relevance of the proposed preoperative prediction model [33,34,35]. Furthermore, there was a lack of information to judge the independence of outcome determination from the knowledge of predictor information. We rated the overall concern for applicability as unclear for all included studies due to the unclear method of outcome determination (transfusion indication) in all studies and the questionable timing of outcome assessment (postoperative interval) in some studies [33,34,35].

The model’s predictive performance varied among the included studies. Due to the clinically significant heterogeneity and the fact that no model was validated more than once in the included studies, a pooled meta-analysis of the predictive performance was not performed.

### 4.2. Relevant Literature

Previously, Dhiman et al. conducted a systematic review of the clinical prediction models for blood transfusion in patients undergoing elective surgery, focusing on their performance and risk of bias [38]. Sixty-six studies (72 developed and 48 externally validated models) published between 1 January 2000 and 30 June 2021 were included. Articles published in non-English languages and those with data containing more than 20% urgent or emergency surgeries were excluded. The majority of studies on blood transfusion prediction models focused on cardiothoracic and general surgery. Most models predicted binary blood transfusion outcomes and red blood cell transfusions. Over half of the models predicted the outcome during the intraoperative and postoperative periods. Logistic regression was the most commonly used method in both developed and validated models. The common predictors for red blood cell (RBC) transfusion included preoperative hemoglobin levels, age, and sex, which were used across various clinical specialties. The models showed moderate to good discriminatory performance, with pooled c-statistic estimates ranging from 0.67 to 0.78. However, most of the prediction models reviewed were found to have a high risk of bias, primarily due to methodological issues in their analysis and poor reporting quality. The authors highlighted the need for better methodological practices and reporting standards in developing and validating these models. Of note, three studies on gynecologic surgery were included, making up 4.6% of all the studies included [33,34,35]. In the present systematic review, we delved into these studies in more detail, examining and reporting their characteristics, risk of bias, applicability concerns, and predictive performance. Additionally, we included two more recently published studies in our review [36,37].

### 4.3. Strengths and Limitations

We clearly defined the review question using the PICOTS framework to ensure clinical relevance. We specifically focused on elective surgery, considered all model development techniques, and emphasized preoperative application. The literature search, screening, and selection process had broad coverage and was overly inclusive and efficient, ensuring that all relevant studies were identified and included. Data extraction was carried out using the CHARMS checklist, and the risk of bias and applicability concerns were evaluated with the PROBAST tool.

However, certain limitations exist due to incomplete reporting of the included studies. The required information for answering PROBAST signaling questions for the assessment of the risk of bias and applicability concern was frequently missing. Without comprehensive and transparent reporting of essential study details, it becomes challenging for the scientific and healthcare community to make an unbiased assessment of the merits and shortcomings of a prediction model study [39]. This issue emphasizes the importance of adhering to the transparent reporting of a multivariable prediction model for individual prognosis or diagnosis (TRIPOD) recommendations when reporting studies on prediction models [40]. Engaging in this practice can assist readers and reviewers in comprehending and critically evaluating published reports. One limitation to also consider is the lack of multiple validations for the models, which prevented us from conducting a pooled meta-analysis to assess predictive performance.

### 4.4. Implications for Research

There is a clear research gap in this field. Future research should prioritize several key areas. Firstly, most of the evaluated models lack external validation. This shortcoming may affect the reliability of the model and limit its generalizability and applicability in different populations and clinical settings. Future studies should pay attention to external validation, including testing the model in different medical centers and populations, testing the model on data that was not involved in its development, and continuously verifying the stability and effectiveness of the model in clinical practice. The predictors and performance estimates synthesized in this review can inform sample size calculations for such studies. Secondly, model developers should focus on developing objective outcome definitions—specifically, transfusion indications—that reflect the actual need for blood transfusion, preferably based on standard or national guidelines, rather than simply recording administered transfusions. In the absence of uniform standards, different hospitals and doctors may make different decisions based on their own experience or inconsistent guidelines, resulting in model prediction bias and incomparable cross-center research results. Thirdly, for the model development studies, model assumptions should be rigorously tested, and non-linear continuous predictors should be handled using methods like restricted cubic splines or fractional polynomials, avoiding the unnecessary categorization of continuous variables [41,42]. Moreover, as EPV ≥ 10 is widely used as a bottom-line criterion for minimizing overfitting, the sample size should be maximized. For smaller EPVs, authors should quantify the degree of model misfit and make optimistic adjustments based on internal validation, shrinking the regression coefficients to reduce bias. In addition, researchers should employ appropriate techniques for handling missing data, such as multiple imputation, rather than relying on complete-case analyses [43,44]. The proper method of candidate predictor selection should also be considered. Furthermore, internal validation methods, such as bootstrapping or cross-validation, are preferable to data-splitting techniques [41,45]. Additionally, comprehensive reporting of both discrimination and calibration measures is essential for a thorough assessment of model performance. To enhance the quality and reliability of future studies, adherence to the TRIPOD guidelines is crucial.

In order to significantly enhance the research contribution in this field, future work should focus on the following points. First, it would be practically useful to clarify the clinical application guidelines of the model to ensure its compatibility with existing processes. Second, it is recommended that a long-term update and maintenance mechanism be established for the model to maintain its effectiveness. Third, cross-center validation in multiple medical centers is encouraged to evaluate the applicability of the model in different patient groups. In addition, detailed operating guidelines should be developed, and clear patient communication strategies should be provided to enhance the model’s practical application value. By addressing these areas, future research can significantly improve the development, validation, and clinical applicability of prediction models for perioperative blood transfusion in gynecologic surgery. This, in turn, could lead to more efficient resource allocation and improved patient outcomes in this important area of perioperative care.

## 5. Conclusions

This systematic review critically evaluates the existing predictive models for perioperative blood transfusion in patients undergoing gynecologic surgery. Currently, there are few models available, and most lack external validation, leading to inconsistent performance. Furthermore, all models are at high risk of bias. Consequently, there is an urgent requirement to develop and validate more robust and widely applicable models. This will hopefully enable the implementation of personalized precision medicine and blood management policies in a timely manner.

## Figures and Tables

**Table 1:** Characteristics of the studies included in the systematic review.

Author, Year	Study Design	Enrollment Period	Study Setting/ Surgical Procedure	Study Region	Participant Characteristics				
					Age	BMI	Surgical Route	Gynecologic Oncology Procedure	Transfusion Rate
Stanhiser, 2017 [33]	Retrospective cohort	1 January 2010–30 June 2014	A single health system/ Various types of gynecological surgery	United States	49.8 (12.7)	30.7 (8.1)	Open:MIS 2025 (16.6):2492 (20.4) (planned)	454 (4.0)	239 (2.0)
Ackroyd, 2018 [34]	Retrospective cohort	2014–2016	The ACS-NSQIP database/ Hysterectomy for ovarian cancer	United States	No transfusion: 59 (17), Transfusion: 62 (17)	No transfusion: overweight & obese 1806 (70.2), Transfusion: overweight & obese 601 (67.6)	No transfusion: Open:MIS 2212 (85.8):367 (14.2), Transfusion: Open:MIS 876 (98.3):15 (1.7)	3470 (100.0)	891 (25.7)
Klebanoff, 2021 [35]	Case-control (nested)	2014–2017	The ACS-NSQIP database/ Myomectomy	United States	No transfusion: 36.6 (6.5), Transfusion: 36.5 (5.6)	No transfusion: 29.0 (6.9), Transfusion: 29.8 (7.1)	No transfusion: Open:MIS 3123 (54.2):2641 (45.8), Transfusion: Open:MIS 551 (88.4):72 (11.6)	0 (0)	623 (9.8)
Walczak, 2021 [36]	Retrospective cohort	1 October 2011–1 October 2017	A large urban nonprofit teaching hospital with over 1000 beds count/ Myomectomy	United States	36 (4.96)	28.26 (9.21)	-	-	7 (7.3)
Hamilton, 2024 [37]	Retrospective cohort	2012–2020	The ACS-NSQIP database/ Laparoscopic myomectomy	United States	No transfusion: ≥40:<40 3910 (35.0):7255 (64.9), Transfusion: ≥40:<40 100 (30.2): 231 (69.8)	No transfusion: 28.5 (7.4), Transfusion: 29.0 (8.3)	-	0 (0)	331 (2.9)

Abbreviation: BMI, body mass index; MIS, minimally invasive surgery; ACS-NSQIP, American College of Surgeons National Surgical Quality Improvement Program.

**Table 2:** Characteristics of the models included in the systematic review.

Author, Year	Modeling Method	Sample Size	Events (%)	No Predictors Cand. Final		EPV or EPP	Selection of Candidate Predictors	Selection of Final Predictors	Number (%) and Handling of Missing Data	Type of Validation	Performance Measures
Stanhiser, 2017 [33]	Logistic regression	12,219 6100	239 (2.0) Missing	22	12	10.9	Based on univariable associations	Backward elimination	*N* (%): Unknown Method: Multiple imputation	Int: Bootstrapping Ext: Temporal	Disc: C-Statistic Internal validation: 0.906 (0.890–0.928) External validation: 0.915 (0.872–0.954) Cal: Calibration plot The calibration curve of the model’s performance showed excellent predictions throughout the range of predicted risks and was accurate through a range of predicted probabilities of 0% to approximately 40% risk of transfusion. Ov: Brier score: 0.017
Ackroyd, 2018 [34]	Logistic regression	2004 1466	Missing Missing	25	8	35.6	Based on univariable associations	Stepwise selection	*N* (%): Unknown Method: No information	Int: None (Apparent performance) Ext: Temporal	Disc: C-Statistic Development: 0.8 (0.78–0.83) External validation: 0.69 (0.66–0.72) Cal: Calibration plot / HL test Calibration plot: High degree of agreement between predicted and actual probabilities HL *p*-value: 0.81 (development) 0.56 (validation)
Klebanoff, 2021 [35]	Logistic regression	6387	623 (9.8)	36	4	17.3	Based on univariable associations	Stepwise selection	*N* (%): Unknown Method: No information	Int: Bootstrapping Ext: None	Disc: AUC graph AUC 0.792 (0.790–0.794) Cal: Calibration plot / HL test Calibration plot: Concordant relationship between the observed incidence and predicted probability of transfusion in the validated model HL *p*-value: 0.68 (development)
Walczak, 2021 [36]	Artificial neural networks	96	7 (7.3)	10	10	0.7	Based on prior knowledge	Other	*N* (%): Unknown Method: No information	Int: Cross-validation Ext: None	Disc: Not evaluated Cal: Not evaluated Ov: sensitivity and overall accuracy
Hamilton, 2024 [37]	Logistic regression	11,498	331 (2.9)	16	4.6	20.7	Based on univariable associations	Unclear	*N* (%): Unknown Method: No information	Int: Bootstrapping Ext: None	Disc: AUC graph 4-parameter model: AUC 0.69 (0.66–0.71) 6-parameter model: AUC 0.78 (0.76–0.80) Cal: Not evaluated

Abbreviation: Cand, candidate; C-statistic, concordance statistic; EPV, event per variable; EPP, event per parameter; HL, Hosmer-Lemeshow test; Int, internal; Ext, external; Cal, calibration; Disc, discrimination; AUC, area under the curve; Ov, overall.

**Table 3:** Risk of bias and applicability assessment of model development studies.

Author, Year	Risk of Bias				Applicability			Overall	
	1. Participants	2. Predictors	3. Outcome	4. Analysis	1. Participants	2. Predictors	3. Outcome	Risk of Bias	Applicability
Stanhiser, 2017 [33]	+	+	?	−	+	+	?	−	?
Ackroyd, 2018 [34]	+	+	?	−	+	+	?	−	?
Klebanoff, 2021 [35]	+	?	?	−	+	+	?	−	?
Walczak, 2021 [36]	+	?	?	−	+	+	?	−	?
Hamilton, 2024 [37]	+	?	?	−	+	+	?	−	?
Note	+	Low risk of bias		−	High risk of bias		?	Unclear	

## Data Availability

No new data were created or analyzed in this study. Data sharing is not applicable to this article.

## Supplementary Materials

The following supporting information can be downloaded at: https://www.mdpi.com/article/10.3390/diagnostics14182018/s1, File S1: Search strategy; File S2: Data extraction form; File S3: Details of risk of bias assessment.
