# Supplementary material for: Prediction Models for Perioperative Blood Transfusion in Patients Undergoing Gynecologic Surgery: A Systematic Review
Source: Diagnostics (Basel). 2024 Sep 12;14(18):2018. doi: 10.3390/diagnostics14182018 (PMC11431761; doi:10.3390/diagnostics14182018)
Supplement: Supplementary file 1 [file diagnostics-14-02018-s001.zip › File S1_Search Strategy.pdf]

Search Strategy

PubMed

Key concept	Search syntax	Records found
GYN surgery	hysterect*[Text Word] OR myomect*[Text Word] OR gynecolog* surg*[Text Word] OR gynaecolog* surg*[Text Word] OR gynecolog* procedure*[Text Word] OR gynaecolog* procedure*[Text Word] OR gynecolog* operation*[Text Word] OR gynaecolog* operation*[Text Word] OR hysterectomy[MeSH Terms] OR hysterectomy, vaginal[MeSH Terms] OR ”uterine myomectomy"[MeSH Terms] OR uterine cervical neoplasms[MeSH Terms] OR endometrial neoplasms[MeSH Terms] OR ovarian neoplasms[MeSH Terms]	340,763
Prediction model	predict*[Text Word] OR model*[Text Word] OR "predict* model"[Text Word] OR "risk score*"[Text Word] OR nomogram*[Text Word] OR “risk adjustment” [Text Word] OR “risk analysis” [Text Word] OR “risk assessment” [Text Word] OR “risk evaluation” [Text Word] OR “diagnostic model”[Text Word] OR “risk factor*”[Text Word] OR “predict* factor*”	7,501,954
Blood loss/Transfusion	"blood loss"[Text Word] OR hemorrhage*[Text Word] OR haemorrhage*[Text Word] OR bleeding[Text Word] OR "blood transfusion"[Text Word] OR hemorrhage[MeSH Terms] OR blood loss, surgical[MeSH Terms] OR postoperative hemorrhage[MeSH Terms]	734,620
Overall	("hysterect*"[Text Word] OR "myomect*"[Text Word] OR ("gynecolog*"[All Fields] AND	4,902

	"surg*"[Text Word]) OR ("gynaecolog*"[All Fields] AND "surg*"[Text Word]) OR ("gynecolog*"[All Fields] AND "procedure*"[Text Word]) OR ("gynaecolog*"[All Fields] AND "procedure*"[Text Word]) OR ("gynecolog*"[All Fields] AND "operation*"[Text Word]) OR ("gynaecolog*"[All Fields] AND "operation*"[Text Word]) OR "hysterectomy"[MeSH Terms] OR "hysterectomy, vaginal"[MeSH Terms] OR "uterine myomectomy"[MeSH Terms] OR "uterine cervical neoplasms"[MeSH Terms] OR "endometrial neoplasms"[MeSH Terms] OR "ovarian neoplasms"[MeSH Terms]) AND ("predict*"[Text Word] OR "model*"[Text Word] OR "predict model"[Text Word] OR "risk score*"[Text Word] OR "nomogram*"[Text Word] OR "risk adjustment"[Text Word] OR "risk analysis"[Text Word] OR "risk assessment"[Text Word] OR "risk evaluation"[Text Word] OR "diagnostic model"[Text Word] OR "risk factor*"[Text Word] OR "predict factor"[Text Word]) AND ("blood loss"[Text Word] OR "hemorrhage*"[Text Word] OR "haemorrhage"[Text Word] OR "bleeding"[Text Word] OR "blood transfusion"[Text Word] OR "hemorrhage"[MeSH Terms] OR "blood loss, surgical"[MeSH Terms] OR "postoperative hemorrhage"[MeSH Terms])	

Embase

Key concept	Search syntax	Records found
GYN surgery	hysterect*[Text Word] OR myomect*[Text Word] OR gynecolog* surg*[Text Word] OR gynaecolog* surg*[Text Word] OR gynecolog* procedure*[Text Word] OR gynaecolog* procedure*[Text Word] OR gynecolog* operation*[Text Word] OR gynaecolog* operation*[Text Word] OR hysterectomy[Emtree] OR myomectomy[Emtree] OR uterine cervix cancer[Emtree] OR endometrium cancer[Emtree] OR ovary tumor[Emtree] OR ovary cancer[Emtree]	675,059
Prediction model	predict*[Text Word] OR model*[Text Word] OR "predict* model"[Text Word] OR "risk score*"[Text Word] OR nomogram*[Text Word] OR “risk adjustment” [Text Word] OR “risk analysis” [Text Word] OR “risk assessment” [Text Word] OR “risk evaluation” [Text Word] OR “diagnostic model”[Text Word] OR “risk factor*” OR “predict* factor*”	10,140,068
Blood loss/Transfusion	"blood loss"[Text Word] OR hemorrhage*[Text Word] OR haemorrhage*[Text Word] OR bleeding[Text Word] OR "blood transfusion"[Text Word] OR bleeding[Emtree]	1,259,552
Overall	hysterect*[Text Word] OR myomect*[Text Word] OR gynecolog* surg*[Text Word] OR gynaecolog* surg*[Text Word] OR gynecolog* procedure*[Text Word] OR gynaecolog* procedure*[Text Word] OR gynecolog* operation*[Text Word] OR gynaecolog* operation*[Text Word] OR hysterectomy[Emtree] OR myomectomy[Emtree]	13,851

	OR uterine cervix cancer[Emtree] OR endometrium cancer[Emtree] OR ovary tumor[Emtree] OR ovary cancer[Emtree] AND predict*[Text Word] OR model*[Text Word] OR "predict* model"[Text Word] OR "risk score*"[Text Word] OR nomogram*[Text Word] OR “risk adjustment” [Text Word] OR “risk analysis” [Text Word] OR “risk assessment” [Text Word] OR “risk evaluation” [Text Word] OR “diagnostic model”[Text Word] OR “risk factor*” OR “predict* factor*” AND "blood loss"[Text Word] OR hemorrhage*[Text Word] OR haemorrhage*[Text Word] OR bleeding[Text Word] OR "blood transfusion"[Text Word] OR bleeding[Emtree]	
