# Supplementary material for: Prediction Models for Perioperative Blood Transfusion in Patients Undergoing Gynecologic Surgery: A Systematic Review
Source: Diagnostics (Basel). 2024 Sep 12;14(18):2018. doi: 10.3390/diagnostics14182018 (PMC11431761; doi:10.3390/diagnostics14182018)
Supplement: Supplementary file 1 [file diagnostics-14-02018-s001.zip › File S2_Data Extraction Form.pdf]

File S2

Data extraction form

- Specific research: author and publication year of the paper, recruitment date of cases, country, number of centers, development or validation (internal/external)
- Surgical details: surgical procedure (open/laparoscopic/robotic/vaginal), duration of surgery, use of special drugs/instruments during the perioperative period
- Pathological features: tumor size, weight, quantity, location
- Patient characteristics: demographic characteristics, past history, comorbidities, preoperative blood indicators, imaging features
- Method: Detailed information on the original dataset (prospective, retrospective [cross-sectional, cohort, case-control])
- Model: The predictive variables included in the model, statistical methods (algorithms) used for model development and internal validation, and the handling of missing data
- The predictive performance of the model: (calibration measurements, such as calibration maps, calibration slopes, etc.) Hosmer Lemeshow test and discriminant measurement, such as C-statistic D-statistic, log rank) results
- Prediction results: blood loss measurement, transfusion requirements
- Data on statistical values of c and their 95% confidence interval
