# Supplementary material for: Prediction Models for Perioperative Blood Transfusion in Patients Undergoing Gynecologic Surgery: A Systematic Review
Source: Diagnostics (Basel). 2024 Sep 12;14(18):2018. doi: 10.3390/diagnostics14182018 (PMC11431761; doi:10.3390/diagnostics14182018)
Supplement: Supplementary file 1 [file diagnostics-14-02018-s001.zip › File S3 Details of risk of bias assessment.pdf]

File 3 Risk of bias judgment for all development (n=6 models) and validation analyses (n=2 models).

Study	Study design	Analysis detail	Participants			Predictors				Outcome							Analysis										Overall judgement
**Development analysis (6 models)**			**1**	**2**	**Judgement**	**1**	**2**	**3**	**Judgement**	**1**	**2**	**3**	**4**	**5**	**6**	**Judgement**	**1**	**2**	**3**	**4**	**5**	**6**	**7**	**8**	**9**	**Judgement**	
Stanhiser, 2017	D	Development of 1 model	+	+	Low	+	?	+	Low	?	+	?	?	?	+	Unclear	-	+	?	+	-	?	+	+	+	High	High
Ackroyd, 2018	D	Development of 1 model	+	+	Low	+	?	+	Low	?	+	?	?	?	+	Unclear	+	?	?	?	-	?	+	-	+	High	High
Klebanoff, 2021	D	Development of 1 model	+	+	Low	+	-	+	Unclear	-	?	+	?	?	+	Unclear	-	?	+	?	-	?	+	+	+	High	High
Walczak, 2021	D	Development of 1 model	+	+	Low	+	?	?	Unclear	?	+	?	?	?	+	Unclear	-	+	+	?	+	?	+	?	?	High	High
Hamilton, 2024	D	Development of model 1	+	+	Low	+	?	-	Unclear	?	+	?	?	?	+	Unclear	+	-	?	?	-	?	-	+	+	High	High
Hamilton, 2024	D	Development of model 2	+	+	Low	+	?	-	Unclear	?	+	?	?	?	+	Unclear	+	-	?	?	-	?	-	+	+	High	High
**Validation of 2 models**																											
Stanhiser, 2017	DV	Validation of developed model	+	+	Low	+	?	+	Low	?	+	?	?	?	+	Unclear	-	+	?	+	NA	?	+	NA	NA	High	High
Ackroyd, 2018	DV	Validation of developed model	+	+	Low	+	?	+	Low	?	+	?	?	?	+	Unclear	+	?	?	?	NA	?	+	NA	NA	High	High

+ = yes/probably yes; - = no/probably no; ? = no information; NA=not applicable
